# Giant urinary bladder stone: A rare case report

**DOI:** 10.1016/j.ijscr.2024.110174

**Published:** 2024-08-14

**Authors:** Ganesh Bhakta Acharya, Suman Baral, Sunil Man Bijukchhe, Sushil Mishra, Krishna Bhusal, Shasi Poudel

**Affiliations:** a Department of Urology, Manipal College of Medical Sciences, Pokhara, Nepal; b Department of Surgery, Mediplus Hospital and Trauma Center, Pokhara, Nepal; c Department of Surgery, Gandaki Medical College, Pokhara, Nepal; d Department of Surgery, Manipal College of Medical Sciences, Pokhara, Nepal; e Department of Emergency Medicine, Lumbini Medical College, Palpa, Nepal; f Department of Emergency Medicine, Fewacity Hospital, Pokhara, Nepal

**Keywords:** Cystoscopy, CT-KUB, Giant urinary bladder stone, Open cystolithotomy

## Abstract

**Introduction:**

Giant urinary bladder stones are rare phenomenon which is associated with chronic urinary infections, intravesical foreign bodies, urethral strictures, bladder diverticula etc.

**Case report:**

A 52-year-old man presented with complaints of severe dysuria, urgency, frequency, suprapubic pain, and pollakuria for the last ten years. Physical examination revealed a palpable suprapubic mass with no obvious flank masses. Ultrasonography of the abdomen and pelvis revealed right-sided gross hydroureteronephrosis and thinning of renal parenchyma along with a hyperechoic structure with posterior acoustic shadowing was noted in the region of the urinary bladder, suggesting a vesical calculus. Plain CT of the kidneys, ureters, and bladder (KUB) confirmed right nephrolithiasis in lower poly calyx and a large vesical calculus (10.6 cm × 8.6 cm x8.8 cm, +1110 HU). Open cystolithotomy with a right-sided double-J “DJ” stent insertion was performed. Post-operative period went uneventful.

**Discussion:**

Giant bladder stones are extremely rare, often resulting from neglected symptoms in otherwise normal individuals. They typically develop over several years and present with symptoms like severe dysuria, urgency, frequency, supra-pubic pain, and hematuria. Diagnosis is made by cystoscopy, ultrasonography and CT-KUB. Treatment includes intracorporeal cystolithotripsy using a laser, ultrasonic lithotripter, or pneumatic lithotripter, depending on availability. Endourologic procedures have been safer and more cost-effective for bladder stones, however, open removal is the treatment of choice for giant bladder stones.

**Conclusion:**

Open cystolithotomy can be performed to remove giant bladder stone with near 100 % stone removal rate.

## Highlights

• Giant bladder stones are associated with chronic urinary infections, intravesical foreign bodies, urethral strictures, bladder diverticula etc.
• Stones present with symptoms like severe dysuria, urgency, frequency, supra-pubic pain, and hematuria
• Diagnosis is made by cystoscopy, ultrasonography and CT-KUB examination
• Treatment includes open cystolithotomy for giant bladder stones.

## 1 Introduction

Urinary bladder calculi account for approximately 5 % of urological stones with multifactorial etiologies such as bladder outlet obstruction, metabolic or genetic diseases, and environmental factors [1]. Chronic urinary infection and intravesical foreign bodies are commonly encountered clinical scenarios that predispose individuals to stone formation. Other causes include abdominal irradiation, bladder augmentation surgery, urethral strictures, schistosomiasis, and bladder diverticula [2,3]. We present a case of a giant bladder calculus, which we believe is the largest ever operated and reported in Nepal to date at our institute. This case has been reported in line of SCARE criteria [4].

## 2 Case report

**Fig. 1:**
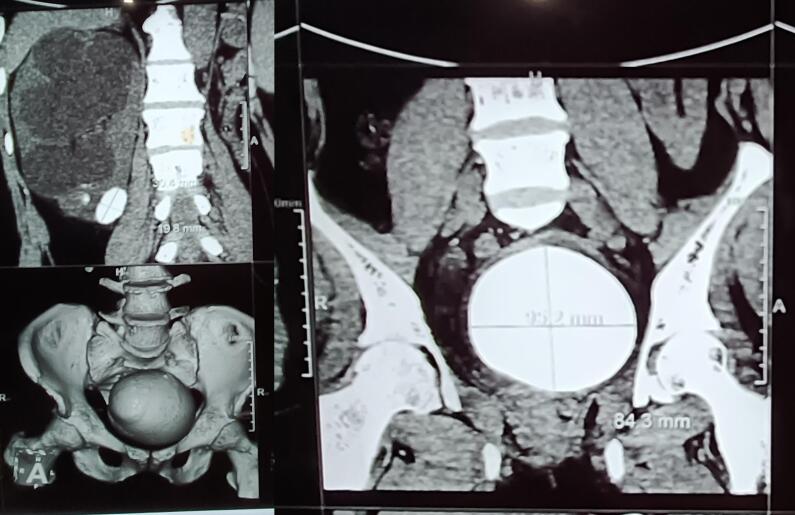
Plain CT of the kidneys, ureters, and bladder (KUB) showing Right Nephrolithiasis in lower poly calyx causing gross hydroureteronephrosis, left renal agenesis, and a large vesical calculus (10.6 cm × 8.6 cm × 8.8 cm, +1110 HU).

**Fig. 2:**
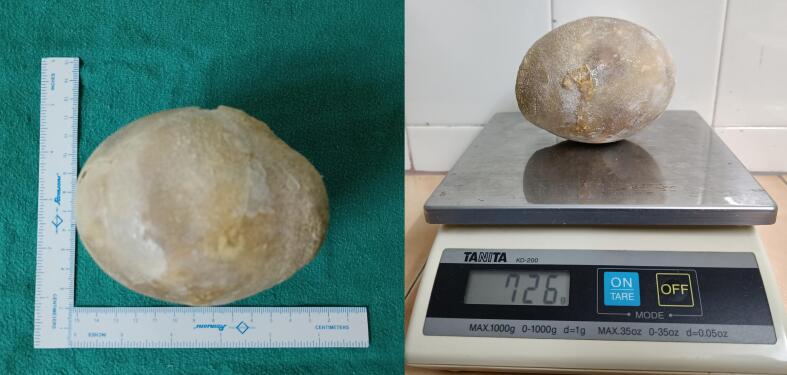
Shows a large bladder stone measuring 11 cm × 9 cm × 8.5 cm and weighing 726 g.

A 52-year-old man presented with complaints of severe dysuria, urgency, frequency, suprapubic pain, and pollakuria for the last ten years. He initially sought relief with over-the-counter medications, which provided temporary remission and delayed further evaluation. In recent months, his symptoms worsened, and the over-the-counter local medications failed to provide relief, prompting him to seek medical attention. Physical examination revealed a palpable suprapubic mass with no obvious flank masses. His routine blood reports were within normal limits, except serum creatinine which was 1.6 mg/dL (normal range: 0.6–1.4 mg/dL). Urine culture showed no growth. Ultrasonography of the abdomen and pelvis revealed right-sided gross hydroureteronephrosis and thinning of renal parenchyma, while the left kidney was not visualized in the left renal fossa. A hyperechoic structure with posterior acoustic shadowing was noted in the region of the urinary bladder, suggesting a vesical calculus. Plain CT of the kidneys, ureters, and bladder (KUB) confirmed right nephrolithiasis in lower poly calyx (few calculi, largest measuring 30 × 22 mm +895 HU) causing gross hydroureteronephrosis, left renal agenesis, and a large vesical calculus (10.6 cm × 8.6 cm × 8.8 cm, +1110 HU) (Fig. 1). Cystoscopy examination showed no infra-vesical obstruction. Open cystolithotomy with a right-sided double-J “DJ” stent insertion was performed after giving Pfannenstiel incision. A large bladder stone measuring 11 cm × 9 cm × 8.5 cm and weighing 726 g was removed (Fig. 2). The post-operative period was uneventful, with improved serum creatinine levels at 1.2 mg/dL. Right renal stone removal was performed six weeks later. Stone analysis revealed a composition of struvite and newberyite. The patient is doing well and remains on regular follow-up.

## 3 Discussion

Giant bladder stones are extremely rare, often resulting from neglected symptoms in otherwise normal individuals which we believe to be applicable in this condition. Despite of the negligence, inaccessibility to the proper health care services also plays a significant role [5] They typically develop over several years and present with symptoms like severe dysuria, urgency, frequency, supra-pubic pain, and hematuria. Secondary bladder stones often develop in patients with infra-vesical obstruction or neurological dysfunction, while primary stones usually present without evidence of uropathy [6]. Most of these stones are composed of calcium oxalate, struvite, calcium phosphate, or ammonium urate. They commonly occur in male patients with potential risk factors such as an enlarged prostate, urethral strictures, neurological disorders, or foreign body in the bladder. The female population accounts for only 5 % of total cases, with risk factors of pelvic surgeries, genital prolapse, and foreign bodies such as genital accessories and Foley catheters [7]. Typically, these are solitary, although 25–30 % of cases present with multiple stones [5]. Nutritional disorders, such as low levels of magnesium, phosphate, vitamin A, and pyridoxin, along with a diet high in carbohydrates and low in protein, have also been linked to endemic stone formation especially in children [5]. However, the data on incidence of the bladder stones is scanty especially in low-income countries including Nepal.

Symptoms include hematuria, urinary retention, and features of recurrent urinary tract infections. Rarely, they present with features of renal failure, and even solitary bladder stones are a rare phenomenon, as most cases present with associated renal or ureteric stones, as in our case [8].

The diagnosis of bladder stones is preferably done by cystoscopy examination of the urinary bladder; however, an X-ray of the abdomen along with ultrasonography will suffice to establish a diagnosis [1]. Similarly, CT KUB has the advantage of diagnosing small stones in the urinary bladder. Low dose (LD) and ultra-low dose (ULD) CT has been shown to have high diagnostic accuracy, sensitivity, and specificity for identifying urinary tract stones with a significant radiation dose reduction in comparison to standard dose CT [9]. The sensitivity of ultrasonography for the diagnosis of bladder stones has been reported to be 20–83 %, with an overall specificity of 98–100 %.

The treatment of bladder stones is usually performed by intracorporeal cystolithotripsy using a laser,ultrasonic lithotripter, or pneumatic lithotripter, depending on availability [10]. The pneumatic lithotripter has the advantage of being cost-effective and widely available. Endourologic procedures have been safer and more cost-effective, and they have almost replaced open stone removal. However, the open method remains the treatment of choice, especially for large bladder stones as those we have encountered in our case [5,6]. Regardless of the technique applied or available, the main goal of treatment is to achieve a complete stone-free rate in the shortest time possible without complications and with a reduced length of hospital stay [6].

## 4 Conclusion

Usually, larger bladder stones are significant clinical findings, and such cases can be effectively managed with open cystolithotomy, which remains a safe procedure with nearly a hundred percent stone clearance rate.

## Consent

Written informed consent was obtained from the patient for publication of this case report and accompanying images. A copy of the written consent is available for review by the Editor-in-Chief of this journal on request.

## Ethical approval

Ethical approval was not mandatory for publication of case reports as per the institutional policy of Manipal College of Medical Sciences, Pokhara.

## Funding

This case report did not receive any specific grant from funding agencies in the public, commercial, or not-for-profit sectors.

## Author contribution

Design and Idea: Ganesh Bhakta Acharya, Suman Baral, Sushil Mishra.

Drafting: Ganesh Bhakta Acharya, Suman Baral, Shasi Poudel, Sunil Man Bijukchhe, Krishna Bhusal.

Final Revision: Ganesh Bhakta Acharya, Suman Baral, Shasi Poudel, Sushil Mishra, Sunil Man Bijukchhe, Krishna Bhusal.

## Guarantor

Suman Baral.

## Research registration number

NA.

## Conflict of interest statement

Authors declare that there are no any conflicts of interest regarding publication of the manuscript.
